# Effect of fluensulfone on different functional genes of root-knot nematode *Meloidogyne incognita*

**DOI:** 10.21307/jofnem-2021-073

**Published:** 2021-08-17

**Authors:** Alkesh Hada, Divya Singh, Kranti Kavalipurapu Veera Venkata Satyanarayana, Madhurima Chatterjee, Victor Phani, Uma Rao

**Affiliations:** 1 Division of Nematology, ICAR-Indian Agricultural Research Institute, New Delhi, India; 2 All India Coordinated Research Project on Nematodes, ICAR-Indian Agricultural Research Institute, New Delhi, India; 3 Department of Agricultural Entomology, College of Agriculture, Uttar Banga Krishi Viswavidyalaya, Dakshin Dinajpur, West Bengal, India

**Keywords:** Chemosensation, Fluensulfone, Metabolism, Neurotransmission, Parasitism, Root-knot nematode

## Abstract

*Meloidogyne incognita* is an obligate plant-parasitic nematode causing serious damage to agricultural crops. Major constraints in nematode management arose due to the limited availability of non-fumigant nematicides in conjunction with the considerable ill effects of fumigants on human and non-target organisms. Recently, fluensulfone has been reported to be an effective non-fumigant nematicide against plant-parasitic nematodes and the model nematode *Caenorhabditis elegans.* The nematicidal efficacy varies according to its concentration at the time of application, exposure timing, nematode species variability, and even across subpopulations within the same species. It interferes with the key physiological processes of nematodes, like motility, behavior, chemosensation, stylet thrusting, infectivity, metabolism, lipid consumption, tissue integrity, oviposition, egg hatching, and survival. However, the molecular basis of these multivariate physiological anomalies is still largely unknown. Quantitative real-time PCR was carried out to understand the acute transcriptional perturbation of 30 functional genes associated with key physiological and life processes in a *M. incognita* population, following exposure of 10, 50, and 100 ppm of fluensulfone for 5 and 10 hr. The chemical treatment resulted in significant downregulation of all the neuropeptidergic genes, with concomitant repression of majority of genes related to chemosensation, esophageal gland secretion, parasitism, fatty acid metabolism, and G-protein coupled receptors. Collectively, the parasitism genes were found to be perturbed at highest magnitude, followed by the GPCRs and neuropeptidergic genes. These results establish the wide ranging effect of fluensulfone on various metabolic and physiological pathways of nematode.

Over 4,100 species of plant-parasitic nematodes (PPNs) pose a major threat to the present day agriculture accounting an estimated yield loss of US$ 173 billion every year (Decraemer and Hunt, 2006). Amongst the top 10 PPN species that cause majority of the economic damage worldwide, root-knot nematodes (RKNs) of genus *Meloidogyne* are considered to be the most severe (Elling, 2013; Jones et al., 2013). The second-stage juveniles (J2s) of RKNs enter the plant roots and develop permanent feeding sites (giant cells) that nurture them for rest of their growth and reproduction (Berg et al., 2009). While doing so, these microscopic animals develop a complex nexus of interactive cross-talks with their hosts and remarkably reprogram the plant cells for their own benefit. A wide array of nematode-derived effect or molecules, viz., the cell-wall modifying enzymes (Mantelin et al., 2017), esophageal gland secretions (Chaudhary et al., 2019; Ding et al., 2000; Huang et al., 2003); neurotransmitters (e.g. acetylcholine, FLPs, NLPs) (Blanchard et al., 2018; Dash et al., 2017; Kumari et al., 2017; Papolu et al., 2013), chemosensory genes (Fleming et al., 2017; Shivakumara et al., 2019), etc. play pivotal roles during this parasitism process to overcome the plant-foisted go/no-go checkpoints. All the key functional genes collectively contribute in shaping a compatible nematode-plant interaction, which ultimately affect the crop yield from agricultural viewpoint.

In spite of the enormous damage caused by the PPNs to agricultural crops, there still remains an acute scarcity of effective and efficacious nematode management option(s). Predominantly, the management of PPNs is traditionally relied on integrated cultural, physical and biological means with use of insecticidal chemicals (Bridge, 1996; Dutta et al., 2019). But, of late, many of the well-known ‘nematicides’ including the fumigants and insecticides are phased out for their undesirable effects on nature (Kim et al., 2017; US-EPA, 2008, 2009). Amongst the novel nematicides, fluensulfone, fluopyram, fluazaindolizine, and tioxazafen have been proven to be highly effective against the PPNs (Faske and Hurd, 2015; Kearn et al., 2014; Lahm et al., 2017; Slomczynska et al., 2014). Considering these four chemicals, fluensulfone has shown excellent results in controlling the nematodes with unique mode of action (MoA), and is being widely used across the globe (Oka, 2014; Oka et al., 2008, 2009, 2012, 2013;). Unlike the fumigants, fluensulfone has very low toxicity toward non-target organisms and it does not emit any volatile organic compound(s) (Ploeg et al., 2019; Waldo et al., 2019). Exposure to this chemical exerts irreversible nematicidal effects affecting the motility, chemosensory perception, stylet thrusting, feeding, moulting, infection potential, oviposition capacity, egg hatching, behaviour, metabolism, lipid consumption, tissue integrity and survival in root-knot, cyst and other nematode species (Kearn et al., 2014, 2017; Oka and Saroya, 2019; Wram and Zasada, 2019). Chemically, fluensulfone [5-chloro-2-(3,4,4-trifluorobut-3-enylsulfonyl)-1,3-thiazole] belongs to heterocyclic fluoroalkenylsulfone group and acts via contact toxicity on the nematodes when directly applied in soil (Kearn et al., 2014). Exposure of PPNs and *Caenorhabditis elegans* against this chemical resulted in non-recoverable paralysis of the worms with characteristic ‘rod-shaped’ body posture, unlike the resultant ‘wavy’ paralysis due to cholinesterase inhibitors (Oka et al., 2009). Behavioral and electrophysiological studies in *C. elegans* following acute and chronic exposure to fluensulfone revealed its effect to be distinct from the organophosphates, carbamates, and macrocyclic lactones (Kearn et al., 2014). The study also demonstrated that the embryo and larval stages of *C. elegans* are more susceptible toward this chemical, and it inhibits the nematode feeding, moulting, and egg hatching possibly by targeting the mitochondrial function (Kearn et al., 2014). Further investigation with plant-parasitic species *Globodera pallida* also revealed that fluensulfone progressively immobilizes the pre-parasitic J2s in a time and concentration dependent fashion, and compromises the internal integrity of the worm (Kearn et al., 2017). All these reports describe the multifaceted effect of fluensulfone on nematode biology and physiology. However, the underlying gene regulation mechanisms causing such physiological anomalies have not been fully understood.

In the present study, we have tested the effect of fluensulfone on expression of 30 functional genes in an Indian subpopulation of the root-knot nematode *M. incognita*. For this purpose, representative genes associated with chemosensation, esophageal gland secretion, nematode parasitism, fatty acid metabolism, β -oxidation, polyunsaturated fatty acid (PUFA) fractionation, neurotransmission, and G-protein coupled receptors (GPCRs) were selected from *M. incognita*. The effects were observed by directly treating the nematode J2s with different concentrations of fluensulfone (10, 50, and 100 ppm), followed by analysis of transcript levels of the respective genes by quantitative real-time PCR (qRT PCR) at two time points (5 and 10 hr post exposure).

## Materials and methods

### Nematode population and drug material

The pure culture of an Indian isolate of *Meloidogyne incognita* race 1 was raised on susceptible tomato plants (*Solanum lycopersicum* cv. Pusa ruby) in a glasshouse at ICAR-Indian Agricultural Research Institute, New Delhi, India. The nematode infected roots were washed free of soil, eggmasses were hand-picked and hatched via ‘modified Baermann’s funnel technique’ (Whitehead and Hemming, 1965). The freshly (within 24 hr) hatched second-stage juveniles (J2s) were used for experimental purpose.

Fluensulfone was procured from Sigma-Aldrich (purity: 99.99%; Sigma-Aldrich, St. Louis, Missouri, USA) only for experimentation and was stored at 4°C in presence of desiccant silica granules. The chemical was then dissolved in an organic vehicle, i.e., dimethyl sulfoxide (DMSO; pure grade) to prepare a carrier solution of 20,000 ppm (1 mg fluensulfone in 50 µL DMSO). The carrier solution was then dissolved in desired quantity of nuclease free water to achieve 10, 50, and 100 ppm of final fluensulfone concentration, to be used for soaking purpose.

### Chemical exposure, RNA extraction, and cDNA preparation

The *M. incognita* J2s were soaked in three concentrations of fluensulfone (10, 50, and 100 ppm) for exposure, and the effect was studied at 5 and 10 hr post soaking. Soaking was continued in dark on a slowly moving rotator at room temperature (~28°C) maintaining three biological replicates; and J2s soaked in 0.05% DMSO (vehicle control) and M9 buffer (1 mM MgSO_4_, 22 mM KH_2_PO_4_, 42.3 mM Na_2_HPO_4_, and 85.6 mM NaCl: 1 L; pH 7.0) for 5 and 10 hr served as controls. Around 5,000 J2s were used for each replication. Following soaking, total RNA was extracted from the J2s with NucleoSpin RNA kit (Macherey-Nagel, Düren, Germany) according to manufacturer’s protocol as described previously (Phani et al., 2018). The RNA was treated with RQ1 RNase-Free DNase (Promega, Madison, WI, USA) to get rid of any genomic DNA contamination. The integrity and quality of RNA was determined by 1% agarose gel and NanoDrop-1000 spectrophotometer (Thermo Fisher Scientific, Waltham, MA, USA). Approximately 500 ng of RNA was reverse transcribed into total cDNA using cDNA synthesis kit (Superscript VILO, Invitrogen, Carlsbad, CA, USA).

### Analysis of mRNA levels by qRT PCR

A total of 30 genes were selected in the present study governing diverse physiological and behavioral activities in *M. incognita*, amongst which 12 genes (*osm-9*, *gpc-1*, *gpa-11*, *gpa-13*, *grk-2*, *fat-6*, *acs-2*, *mdt-15*, *nhr-49*, *elo-2*, *ech-5*, and *ech-6*) were identified and cloned from *M. incognita* for the first time (Table 1) (Ashrafi, 2007; Bargmann, 2006; Jansen et al., 2002; Lans and Jansen, 2007; Wang et al., 2017). For this, comparative genomics were used to putatively identify the *M. incognita* orthologs of the previously unannotated genes using *C. elegans* genomic database as query (Supplementary Table 1). The respective protein sequences (of *C. elegans* genes) were retrieved from WormBase (Version: WS260) database, and were BLAST searched in WormBase Parasite (http://parasite.wormbase.org/Tools/Blast) and INRA database (http://www6.inra.fr/meloidogyne_incognita/Genomic-resources2/Blast) to fetch the translated nucleotide sequences in *M. incognita*. Identity of the exact putative gene hits in *M. incognita* was deputed from the sequences with smallest expect value and large bit score. The sequences were then checked for presence of corresponding conserved domains and specific primers were designed to amplify the genes from *M. incognita* cDNA. The amplified products were then cloned, sequenced via Sanger sequencing for reconfirmation, and the sequences were submitted to NCBI GenBank (Table 1).

**Table 1.** List of primers used in this study.

Sl	Primers name	Gene name (accession number)	Primers Sequences (5′–3′)	Tm (°C)	Purpose
1	RT miODR-1 F	*Mi-odr-1* (MG780832)	GAACCACGCTCTTACGATTC	60°C	qRT PCR
	RT miODR-1 R		CTCCAGAAGCGACCATGTA		
2	RT miODR-3 F	*Mi-odr-3* (MG780833)	CTGGCTATAGACCCACAGAA	60°C	qRT PCR
	RT miODR-3 R		GAACGTTGTCCACCTACATC		
3	RT miTAX-2 F	*Mi-tax-2* (MG780834)	GAGGGAATATCCTGAAGCG	60°C	qRT PCR
	RT miTAX-2 R		CCTGATTCCACTGTTCTGG		
4	RT miTAX-4 F	*Mi-tax-4* (MG780835)	GTGAAGTTCCTTGGCCTAT	60°C	qRT PCR
	RT miTAX-4 R		CAGAGCTAGCAATCATACTC C		
5	RT miOSM-9 F	*M. incognita osm-9* (MT676864)	CATGCCTGAAGATTGGGAAG	60°C	Cloning and qRT PCR
	RT miOSM-9 R		GGTCAGGATTAGCACCATAC		
6	RT miGPC-1 F	*M. incognita gpc-1* (MT676857)	CAACTTCGACAAGAGGCAA	60°C	Cloning and qRT PCR
	RT miGPC-1 R		CCCTATTGGTCCGGTAATTAAG		
7	RT mi GPA-11F	*M. incognita gpa-11* (MT676854)	CGACTACGGGTATTTGTGAC	60°C	Cloning and qRT PCR
	RT miGPA-11 R		GAAAGTTCGGCTACGAACAG		
8	RT miGPA-13 F	*M. incognita gpa-13* (MT676855)	GCTGAAACTTCAGAGGATGG	60°C	Cloning and qRT PCR
	RT miGPA-13 R		GCTGGTATTCACGAGAACG		
9	RT miGRK-2 F	*M. incognita grk-2* (MT676858)	CTCAATTCTACGCAGCAGAG	60°C	Cloning and qRT PCR
	RT miGRK-2 R		GGTGCCATATAGCCAACAG		
10	RT miXYL-1 F	*Mi-xylanase* (AF224342)	GGGATTAGTTGCATACAGTTAAGATAAG	60°C	qRT PCR
	RT miXYL-1 R		GCTGTTCGAGTAAGCAGTAGAG		
11	RT miXYL-3 F	*Mi-xylanase* (EU475876)	GGAACTTCCGGTAAACCCTA	60°C	qRT PCR
	RT miXYL-3 R		GACCGGACTGATGTGTTATC		
12	RT miENG-1 F	*Mi-β*-*1,4-endoglucanase* (AF100549)	ACCGAGCAACTCACAAAC	60°C	qRT PCR
	RT miENG-1 R		GGCATTGCTACCCGTATTT		
13	RT miPEL F	*Mi-pectatelyase* (AF527788)	CGAATAACGACGAAGAGGAC	60°C	qRT PCR
	RT miPEL R		AGAACCGACAACCCTACA		
14	RT miMSP-20 F	*Mi-msp-20* (AY134439)	TGGTGACGAACGCACACCTACATA	60°C	qRT PCR
	RT miMSP-20 R		GCGCTGTCTTTGACCATTTGCTCT		
15	RT miMSP-33 F	*Mi-msp-33* (AY142118)	GTGGCCTCCTTTGCTTGGACATTT	60°C	qRT PCR
	RT miMSP-33 R		CATCACCTCCAATTACTCCGGGTT		
16	RT miFAT-6 F	*M. incognita fat-6* (MT676846)	GAATTACTGCAGGTCCTCAC	60°C	Cloning and qRT PCR
	RT miFAT-6 R		CATCAGTGTCAGTCCACTTG		
17	RT miACS-2 F	*M. incognita acs-2* (MT676837)	CCCACCAGAGGAAAGGATAA	60°C	Cloning and qRT PCR
	RT miACS-2 R		CGCTCCTATACCAACGATCT		
18	RT miMDT-15 F	*M. incognita mdt-15* (MT676859)	CAGCTATGCCACCAGGTTT	60°C	Cloning and qRT PCR
	RT miMDT-15 R		CCTAATCCTCCTCCACCATTTC		
19	RT miNHR-49 F	*M. incognita nhr-49* (MT676861)	GCCAATGAGGCAATTAGAG	60°C	Cloning and qRT PCR
	RT miNHR-49 R		CCTCGTCTACCGGTTTAGAT		
20	RT miELO-2 F	*M. incognita elo-2* (MT676842)	CAAACGGATATTGGGTCTGG	60°C	Cloning and qRT PCR
	RT miELO-2 R		GGCGTGATAGGGTAGGAATA		
21	RT miECH-5 F	*M. incognita ech-5* (MT676839)	CACTAGGTGGAGGACTAGAA	60°C	Cloning and qRT PCR
	RT miECH-5 R		CTTGGGAGTCTTTGAGTTCC		
22	RT miECH-6 F	*M. incognita ech-6* (MT676840)	CGAGTCTTCTGGAAGGGATA	60°C	Cloning and qRT PCR
	RT miECH-6 R		GCTTAGATGGCAAAGAGGTC		
23	RT miFLP-12 F	*Mi-flp-12* (AY804187)	TGAGGAAGCGGCCCGATAGTTCTT	60°C	qRT PCR
	RT miFLP-12 R		GGATGAAGAAATGCTTGGACGAGT		
24	RT miFLP-14 F	*Mi-flp-14* (AY907829)	GCGAGTCCATGTGTAGCAGCTAAT	60°C	qRT PCR
	RT miFLP-14 R		GGGAGATGAAGAACGTTTACTACTTTGCC		
25	RT miFLP-16 F	*Mi-flp-16* (EU549831)	GGCAATATTCACGAGACTGGCAAC	60°C	qRT PCR
	RT miFLP-16 R		GGCCATTCAATGCCTGAAGAGGG		
26	RT miFLP-18 F	*Mi-flp-18* (AY729022)	AGGATGACTTATTGCGCCAGGA	60°C	qRT PCR
	RT miFLP-18 R		TTCCTTTACCGAATCTGAGCACGC		
27	RT miACE-1 F	*Mi-ace-1* (AF075718)	CTCCTTGTTCTGAGGATTGTCT	60°C	qRT PCR
	RT miACE-1 R		TTTATGGAGGTGGATTCTGGAG		
28	RT miACE-2 F	*Mi-ace-2* (AF495588)	AGATGTGGAATCCGCCTAATG	60°C	qRT PCR
	RT miACE-2 R		TTTACTCGGGCTCTCCTTCT		
29	RT miNLP-3 F	*Mi-nlp-3* (KY054882)	TCCACAATTTGTGCCAGGTC	60°C	qRT PCR
	RT miNLP-3 R		TTGTGGGGCTGATCGTTTTC		
30	RT miNLP-12 F	*Mi-nlp-12* (KY054885)	GATAGGCGAAAGCGGACTATT	60°C	qRT PCR
	RT miNLP-12 R		CAAGGGCCTGTAATCTCTCTTT		
31	RT mi18S rRNA F	*Mi-18S rRNA* (HE667742)	TCAACGTGCTTGTCCTACCCTGAA	60°C	qRT PCR
	RT mi18S rRNA R		TGTGTACAAAGGGCAGGGACGTAA		
32	RT mi actin F	*Mi-actin* (BE225475)	TGACTCTGGAGATGGTGTTACG	60°C	qRT PCR
	RT mi actin R		GTGATGACTTGACCGTCAGGC		

**Table S1.** Homology parameters of unannotated genes used in this study.

Sl	Primers name	Gene name (accession number)	Primers Sequences (5′–3′)	Amplicon length (bp)	Identity (%)	Score	E-value	Purpose
1	RT miOSM-9 F	*M. incognita osm-9* homologue	CATGCCTGAAGATTGGGAAG	150	100	124	3.6E‒62	qRT PCR
	RT miOSM-9 R		GGTCAGGATTAGCACCATAC					
2	RT miGPC-1 F	*M. incognita gpc-1* homologue	CAACTTCGACAAGAGGCAA	117	100	199	1.4E‒18	qRT PCR
	RT miGPC-1 R		CCCTATTGGTCCGGTAATTAAG					
3	RT mi GPA-11F	*M. incognita gpa-11* homologue	CGACTACGGGTATTTGTGAC	143	100	58	8.2E‒23	qRT PCR
	RT miGPA-11 R		GAAAGTTCGGCTACGAACAG					
4	RT miGPA-13 F	*M. incognita gpa-13* homologue	GCTGAAACTTCAGAGGATGG	112	100	194	1.1E‒17	qRT PCR
	RT miGPA-13 R		GCTGGTATTCACGAGAACG					
5	RT miGRK-2 F	*M. incognita grk-2* homologue	CTCAATTCTACGCAGCAGAG	124	100	202	4.4E‒19	qRT PCR
	RT miGRK-2 R		GGTGCCATATAGCCAACAG					
6	RT miFAT-6 F	*M. incognita fat-6* homologue	GAATTACTGCAGGTCCTCAC	162	100	306	1.9E‒33	qRT PCR
	RT miFAT-6 R		CATCAGTGTCAGTCCACTTG					
7	RT miACS-2 F	*M. incognita acs-2* homologue	CCCACCAGAGGAAAGGATAA	200	100	356	4.2E‒40	qRT PCR
	RT miACS-2 R		CGCTCCTATACCAACGATCT					
8	RT miMDT-15 F	*M. incognita mdt-15* homologue	CAGCTATGCCACCAGGTTT	100	100	105	2.5E‒5	qRT PCR
	RT miMDT-15 R		CCTAATCCTCCTCCACCATTTC					
9	RT miNHR-49 F	*M. incognita nhr-49* homologue	GCCAATGAGGCAATTAGAG	179	100	294	9.4E‒32	qRT PCR
	RT miNHR-49 R		CCTCGTCTACCGGTTTAGAT					
10	RT miELO-2 F	*M. incognita elo-2* homologue	CAAACGGATATTGGGTCTGG	148	100	64	2.2E‒26	qRTPCR
	RT miELO-2 R		GGCGTGATAGGGTAGGAATA					
11	RT miECH-5 F	*M. incognita ech-5* homologue	CACTAGGTGGAGGACTAGAA	130	100	209	4.7E‒20	qRT PCR
	RT miECH-5 R		CTTGGGAGTCTTTGAGTTCC					
12	RT miECH-6 F	*M. incognita ech-6* homologue	CGAGTCTTCTGGAAGGGATA	193	100	342	1.3E‒36	qRT PCR
	RT miECH-6 R		GCTTAGATGGCAAAGAGGTC					

Quantitative real-time PCR (qRT PCR) was carried out to analyze the expression pattern of the genes in *M. incognita* J2s after exposing them against fluensulfone. The qRT PCR was performed in a Realplex2 thermal cycler (Eppendorf, Hamburg, Germany) using SYBR Green Supermix Kit (Eurogentec, Liege, Belgium) (Phani et al., 2018). Reaction mixture for each sample contained a final volume of 10 µL comprising of 5 µL of SYBR Green PCR Master mix, 750 nM of each primers and 1.5 ng of cDNA. Gene expression level was normalized using two constitutively expressed *M. incognita* genes, *18S rRNA* (HE667742) and *actin* (BE225475). For each of the analyzed genes, three biological and three technical replicates were used, data were analyzed by 2^(‒ΔΔCt) method (Livak and Schmittgen, 2001), and fold change expression values were subjected to Student’s *t* test for determining the statistical significance (*p* = 0.05, 0.01).

## Results

### Gene amplification

A total of 12 genes (out of 30) were identified and cloned in the present study for qRT PCR purpose, which were previously unannotated in *M. incognita* (Supplementary Table 1). For this, reciprocal best hit approach was optimized at ≥30% identity, ≥50% query coverage, and <10^‒5^ E-value for identification of the gene orthologs. Thereafter in silico validation and analyses confirmed the presence of respective conserved motifs in the sequences; and were submitted to NCBI GenBank (Table 1). Owing to the experimental integrity for qRT PCR purpose, concentration of fluensulfone and time of exposure was combined in such way so that no worm dies, and nematicidal effect was microscopically assessed by behavioral changes, immobility and mortality in the *M. incognita* J2s (Supplementary Table 2).

**Table S2.** Assessment of mortality of *M. incognita* J2s at variable concentrations of fluensulfone and time of exposure.

	Nematode mortality (%)						
Fluensulfone concentration (ppm)	1hr	2.5hr	5hr	7.5hr	10hr	24hr	Remarks
100	0.86	3.47	8.62	18.51	27.78	100	Reduced motility and agility observed 0.5hr onward the drug treatment
75	0	1.74	4.35	8.62	18.18	100	Reduced motility and agility observed 1hr onward the drug treatment
50	0	1.74	8.08	13.79	13.79	100	Reduced motility and agility observed 1hr onward the drug treatment
25	0	0.86	2.59	8.62	11.01	90.74	Reduced motility and agility observed 4hr onward the drug treatment
10	0	0	0	2.59	6.03	75.92	Reduced motility and agility observed 4hr onward the drug treatment
5	0	0	0	1.74	1.74	43.48	Reduced motility and agility observed 7hr onward the drug treatment
1	0	0	0	0	1.74	29.57	Reduced motility and agility in few worms observed 7–8hr onward the drug treatment
Vehicle control (0.05% DMSO)	0	0	0	0	0	0	No change in motility and agility

### Effect on chemosensory and GPCR genes

**Figure 1:**
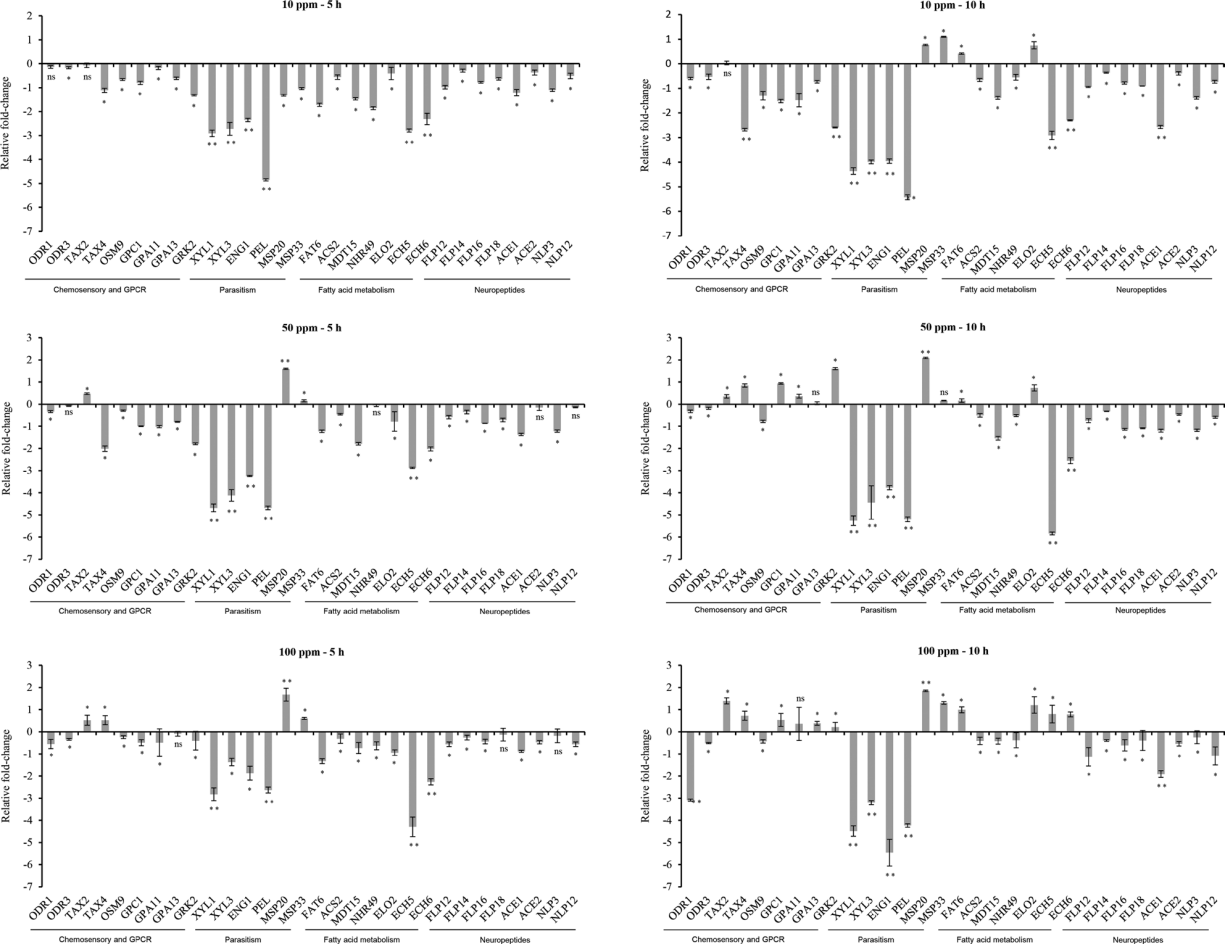
Relative fold-change expression of genes related to nematode chemosensation, GPCRs, parasitism, fatty acid metabolism, and neurotransmission in *M. incognita* J2s after treatment with 10, 50, and 100 ppm of fluensulfone for 5 and 10 hr. Each bar represents the log2-transformed mean ± SE (*n* = 3). Asterisk (*) indicates significant differential expression (*p < 0.05; **p < 0.01; ns = non-significant), analyzed by one-tailed *t*-test.

The DMSO (vehicle) control and M9 (buffer) control were insignificantly different, and the data representing comparative fold change expression between treatments and M9 buffer control were presented here. A total of nine chemosensory and GPCR genes (*odr-1*, *odr-3*, *tax-2*, *tax-4*, *osm-9*, *gpc-1*, *gpa-11*, *gpa-13*, and *grk-2*) were taken in the present study to check their expression level after the J2s being treated with different concentrations of the chemical. The exposure of J2s for 5 and 10 hr against all three fluensulfone concentrations resulted in downregulation of both the *odr* genes in a range of ‒0.08 to ‒3.08 folds (Fig. 1). In contrast, *tax-2* and *tax-4* were up- and downregulated against exposure to different time-concentration combinations (Fig. 1). *osm-9* was downregulated at both the time points against exposure of all the three concentrations. However, *gpc-1*, *gpa-11* and *gpa-13* and *grk-2* showed similar type of transcriptional perturbation with downregulation at 5 hr post exposure for all three concentrations; but upregulated at 10 hr against 50 and 100 ppm of fluensulfone.

### Effect on parasitism genes

The parasitism genes studied here included the *M. incognita* effectors encoding cell wall modifying enzymes (CWMEs) and the esophageal gland secreted proteins. The genes encoding xylanase (*xyl-1* and *xyl-3*), β-1,4-endoglucanase (*eng-1*) and pectatelyase (*pel*) were downregulated at both the time points against exposure of 10, 50, and 100 ppm of fluensulfone (Fig. 1). In contrast, *msp-20* and *msp-33* showed downregulation only at 5 hr post exposure against 10 ppm of fluensulfone, but were upregulated at all other time-concentration combinations (Fig. 1). However, the transcriptional perturbation level of *xyl-1*, *xyl-3*, *eng-1* and *pel* was greater than *msp-20* and *msp-33*.

### Effect on fatty acid metabolism genes

The fatty acid desaturase gene *fat-6* was downregulated at 5 hr post treatment against exposure to all the concentrations in a range of ‒1.23 to ‒1.72 folds; but was upregulated at 10 hr post treatment for all the concentrations to the tune of 0.16–0.99 folds (Fig. 1). The genes governing fatty acid elongation (*elo-2*) also showed similar type of transcriptional perturbation as of *fat-6* (Fig. 1). However, the genes involved in β-oxidation (*acs-2*, *mdt-15*, *nhr-49*, *ech-5*, and *ech-6*) processes were mostly downregulated after treatment at 10, 50, and 100 ppm for 5 and 10 hr, except slight upregulation was observed for *ech-5* (0.80 fold) and *ech-6* (0.78 fold) at 10 hr post exposure against 100 ppm of fluensulfone.

### Effect on neuropeptidergic genes

Here, three types of neuropeptidergic genes representing acetylcholine, FMRFamide like peptides (FLPs) and neuropeptide like protein (NLPs) peptides were studied. Interestingly, all the genes were downregulated in the J2s at both the time points for all the fluensulfone concentrations. In the FLP category, *flp-12*, *flp-14*, *flp-16*, and *flp-18* were downregulated in a range of ‒0.13 to ‒1.14 folds; along with the acetylcholinesterase genes *ace-1* and *ace-2* (Fig. 1). Similarly, expression of the NLP genes, *nlp-3* and *nlp-12* was repressed for all the time-concentration combinations in a range of ‒0.14 to ‒1.39 folds (Fig. 1).

## Discussion

Here, we have determined the deviation of transcript abundance of 30 functional genes related to different physiological processes in *M. incognita*. The effect was determined after treating the nematode J2s with fluensulfone, an olefinic nematicidal compound of 1,3-thiazole class. The chemical exposure was achieved by direct soaking of the worms in 10 ppm (~34.28 µM), 50 ppm (~171.40 µM), and 100 ppm (~342.80 µM) of fluensulfone solution for 5 and 10 hr, and the toxicity effects were ascertained at transcriptional level by qRT PCR. Treatment of *M. incognita* J2s with fluensulfone largely brought a downregulated gene response of the tested genes; however, considerable variability can be observed with regard to this statement depending upon the gene classes and time-concentration combinations. Notably, all the neuropeptidergic genes were downregulated to variable extent for different time-concentration combinations. To the best of our knowledge, this is the first study deciphering the effect of fluensulfone on transcriptional perturbation of genes related to some vital physiological processes in any plant-parasitic nematode.

A considerable disparity lies in the concentrations of fluensulfone that bring nematicidal effect in the free-living species *C. elegans* and other plant-parasitic nematodes (Kearn, 2015; Oka, 2014; Oka et al., 2009). Oka et al. (2009) showed that fluensulfone exerts irreversible nematicidal activity in the *M. javanica* J2s with exposure of 12–48 hr against ≥3.4 μM concentration; and 3.4 µM fluensulfone resulted in chronic, non-spastic paralysis in the J2s with ≥80% mortality at 48 hr. The behavioral signs of nematicidal effect included uncoordinated body movement, reduced locomotion and pharyngeal pumping, defected host recognition, and characteristic body posture (Kearn, 2015; Oka and Saroya, 2019; Oka et al., 2009). However, the nematicidal effect of fluensulfone largely depends on concentration of the chemical, exposure timing, target nematode species, and even across population differences within the same species (Oka and Saroya, 2019; Shirley et al., 2019). In view of achieving the acute nematicidal response, three different concentrations (10, 50, and 100 ppm) of fluensulfone were used in the present investigation to expose the *M. incognita* J2s for relatively shorter time periods (5 and 10 hr). The time-concentration combinations kept the worms alive but exhibited deranged behaviour; and the effect was studied as transcriptional perturbation of the functional genes. The genes (studied here) were chosen for their involvement in diverse physiological processes and key life functions in *M. incognita*, such as chemosensation, neurotransmission, fatty acid metabolism, esophageal gland secretion, and parasitism (Bargmann, 2006; de Almeida Barros et al., 2012; Holden-Dye and Walker, 2011; Perry, 1996; Quentin et al., 2013; Vanholme et al., 2004; Watts and Ristow, 2017).

In *M. incognita*, *odr-1* mRNA was localized in the cell bodies of amphidial neurons and phasmids; and RNAi mediated knockdown of *odr-1*, *odr-3*, *tax-2*, and *tax-4* resulted in defective chemotaxis toward volatile and non-volatile compounds (Shivakumara et al., 2019). Major downregulation of all these genes clearly substantiates the fact that the nematode sensory perception was disturbed in the presence of fluensulfone, which was also recorded previously (Kearn et al., 2014; Oka and Saroya, 2019). However, *tax-2* and *tax-4* was upregulated at higher concentrations for longer time of exposure, which can be attributed by some possible feedback mechanism(s) through which the organism try to restore the physiological setbacks (Swain et al., 2010). The OSMotic avoidance abnormal family member 9 (OSM-9) protein coding gene, *osm-9* is expressed in the sensory neurons being involved in taste adaptation, sensory plasticity, and detection of odorants in *C. elegans* (Jansen et al., 2002). In the present study, *osm-9* showed considerable downregulation for all the treatments. The sensory specific G-protein γ subunit, *gpc-1*; G-protein subunits *gpa-11* and *gpa-13*; and the GPCR kinase *grk-2* showed downregulation at 5 hr post exposure for all the three concentrations, but was slightly upregulated for 50 and 100 ppm concentrations at 10 hr. Previous results have shown that *gpa-11* and *gpa-13* regulate life span and navigation in *C. elegans* (Bargmann, 2006; Lans and Jansen, 2007); and *grk-2* loss-of-function strains were egg laying-defective having low levels of serotonin or 5-hydroxytryptamine (5-HT) (Wang et al., 2017). Studies with 5-HT receptor antagonist on *G. pallida* and *C. elegans* indicated possible interaction of fluensulfone with 5-HT signaling to affect stylet thrusting and pharyngeal pumping (Kearn et al., 2014), and this interaction may be interfered by GPCR kinase *grk-2* activity. Perturbation of all these GPCR receptors and subunits possibly hacked the normal physiological processes leading to abnormalities in nematode’s feeding, way-finding, egg laying, and development. However, the differential transcriptional regulation varying with chemical concentration and time of exposure, as observed here, may be intervened by a network of protective and compensatory mechanisms to mitigate the toxicological effects (Bundy et al., 2008; Swain et al., 2010).

Previous studies showed that exposure of *M. incognita* and *M. javanica* to sublethal concentrations of fluensulfone reduced the number of juveniles attracted to root tips resulting in reduced galling (Oka and Saroya, 2019). The esophageal gland specific genes and CWMEs accredit the host finding, migration inside root, development and maintenance of feeding site in *Meloidogyne* spp. (Ding et al., 2000; Shivakumara et al., 2017). As observed here, suppression of all the CWME genes by fluensulfone may collectively result in reducing the host finding and parasitic potential of the nematode species. However, the *msp* genes tested here were only downregulated at 5 hr post exposure to 10 ppm of the chemical; but were upregulated in all other time-concentration combinations that might be due to complex crosstalk between the pharyngeal gland genes and CWMEs shaping the nematode’s parasitic potential (Shivakumara et al., 2016, 2017). Fluensulfone treatment also affects the fatty acid metabolism, β-oxidation, and PUFA fractionation processes in nematodes resulting in elevated lipid reserve in the treated worms (Kearn et al., 2017). In *C. elegans*, *fat-6* encodes for stearoyl-coA desaturases that catalyze biosynthesis of monounsaturated fatty acids (MUFAs) from saturated fatty acids and is required for large-sized lipid droplet formation (Brock et al., 2007; Shi et al., 2013). The MUFAs are the most abundant constituent of phospholipids and triglycerides (Enoch et al., 1976), and are important mediators for membrane fluidity and signal transduction (Ntambi, 2004). Downregulation of *fat-6*, as seen in the present study, may result in decreased utilization of the stored fat reducing the phospholipid and triglyceride formation. Similar suppression of fatty acid elongation gene *elo-2* might also aid in reducing the PUFA fractionation into these products (Kniazeva et al., 2003). However, for all the three concentrations *fat-6* and *elo-2* were upregulated at 10 hr post exposure. This variability may be brought by the complex nexus of gene regulation pattern underlying the fat metabolism process and the response of fat metabolism pathways to environmental toxicants (Brock et al., 2007; Hermsen et al., 2012). Further attenuation of *acs-2*, *mdt-15*, *nhr-49*, *ech-5*, and *ech-6* genes signifies the possible disruption of β-oxidation process (Ashrafi, 2007). As a consequence, the fluensulfone treated worms possibly possess an elevated level of lipid reserve for inability to breakdown and utilize fat simultaneously affecting the ion channel regulation and membrane transfer activity. The impaired energy metabolism (Kearn, 2015) thus plausibly a resultant effect of reduced phospholipid and triglyceride content at cellular level.

The neuropeptides, acetylcholine, FLPs, and NLPs coordinate many crucial aspects of nematode physiology and behaviour (McCoy et al., 2014; McVeigh et al., 2006, 2008; Rand, 2007). In the present study, notably all the neuropeptidergic genes were repressed after fluensulfone treatment. FLP-12, FLP-14, FLP-16, and FLP-18 were identified in root-knot nematodes to coordinate host recognition, parasitism, and development (Kumari et al., 2017; Papolu et al., 2013). Two neuropeptide like protein coding genes, *nlp-3* and *nlp-12* were found to coordinate locomotion, development, and parasitic ability in *M. incognita* (Dash et al., 2017). Further, *in planta* RNAi silencing of *M. incognita* acetylcholinesterase genes, *ace-1* and *ace-2*, also resulted in reduced galling and parasitism (Cui et al., 2017). These results suggest that fluensulfone might impair the normal activity of these neuropeptides leading to defective developmental, host recognition, locomotion, and parasitism in the nematode. Besides, *ace-1* and *ace-2* encode for class A and class B acetylcholiesterases, and also regulate nematode locomotion (Combes et al., 2003). Hence, the data suggest that fluensulfone possibly act on acetylcholinesterase, but how it differs from the activity of organophosphates and carbamates is yet to be understood.

In conclusion, our results validate that the nematicidal effects of fluensulfone modulate and impair several checkpoints of nematode biology including chemosensation, esophageal gland secretion, fatty acid metabolism, β-oxidation, polyunsaturated fatty acid (PUFA) fractionation, and neurotransmission at transcriptional level. The observed changes in neuropeptidergic gene expression suggest that the chemical interferes with the regular functioning of neuropeptides, which might be a major cause behind the biological anomalies. Additionally, possibilities of engagement of single gene in different biological functions cannot also be overruled (Chew et al., 2018; Shivakumara et al., 2016; Zhang et al., 2013), which can be true for fluensulfone treatment. However, the study does not include any comparison of effects of fluensulfone with other groups of chemical nematicides, and all the possible molecular targets (studied here) affected by the chemical have been discussed. Presumably, the multidimensional effect of this nematicide is achieved by direct action on the genes and/or pathways governing various physiological functions; or its action on the regulatory receptors and neuropeptides creating a functional imbalance of the downstream gene pool in an anti-narrow direction.
